# Association of Fine Particulate Matter Constituents with the Predicted 10-Year Atherosclerotic Cardiovascular Disease Risk: Evidence from a Large-Scale Cross-Sectional Study

**DOI:** 10.3390/toxics11100812

**Published:** 2023-09-26

**Authors:** Sheng Wang, Ge Zhao, Caiyun Zhang, Ning Kang, Wei Liao, Chongjian Wang, Fuwei Xie

**Affiliations:** 1 Key Laboratory of Tobacco Chemistry, Zhengzhou Tobacco Research Institute of CNTC, Zhengzhou 450003, China; wangs@ztri.com.cn (S.W.); zhaogztri@163.com (G.Z.); 2 Department of Epidemiology and Biostatistics, College of Public Health, Zhengzhou University, Zhengzhou 450001, China; zhangcaiyun1017@163.com (C.Z.); kangning97@163.com (N.K.); wliaotr@163.com (W.L.)

**Keywords:** PM_2.5_, PM_2.5_ constituents, BC, ASCVD, rural area

## Abstract

Little is known concerning the associations of fine particulate matter (PM_2.5_) and its constituents with atherosclerotic cardiovascular disease (ASCVD). A total of 31,162 participants enrolled from the Henan Rural Cohort were used to specify associations of PM_2.5_ and its constituents with ASCVD. Hybrid machine learning was utilized to estimate the 3-year average concentration of PM_2.5_ and its constituents (black carbon [BC], nitrate [NO_3_^−^], ammonium [NH_4_^+^], inorganic sulfate [SO_4_^2−^], organic matter [OM], and soil particles [SOIL]). Constituent concentration, proportion, and residual models were utilized to examine the associations of PM_2.5_ constituents with 10-year ASCVD risk and to identify the most hazardous constituent. The isochronous substitution model (ISM) was employed to analyze the substitution effect between PM_2.5_ constituents. We found that each 1 μg/m^3^ increase in PM_2.5_, BC, NH_4_^+^, NO_3_^−^, OM, SO_4_^2−^, and SOIL was associated with a 3.5%, 49.3%, 19.4%, 10.5%, 21.4%, 14%, and 28.5% higher 10-year ASCVD risk, respectively (all *p* < 0.05). Comparable results were observed in proportion and residual models. The ISM found that replacing BC with other constituents will generate the greatest health benefits. The results indicated that long-term exposure to PM_2.5_ and its constituents were associated with increased risks of ASCVD, with BC being the most attributable constituent.

## 1. Introduction

As the primary cause of death and disease burden worldwide, cardiovascular diseases (CVDs) are the most widespread chronic diseases globally [1,2]. In China, there were nearly 94 million CVD cases, 3.97 million all-cause deaths were attributed to CVDs in 2016, and the annual mortality increased by 58.17% from 1990 (2.51 million) to 2016 (3.97 million) [3]. As the leading cause of CVDs, atherosclerotic cardiovascular disease (ASCVD) was responsible for approximately 17.8 million mortalities worldwide in 2017 and 2.4 million deaths in China in 2016, and the burden of disease caused by it has been increasing continuously recently [3,4,5]. Therefore, it is crucial to conduct research on risk factors for ASCVD to establish effective strategies for ASCVD prevention.

PM_2.5_ (fine particulate matter with a diameter ≤2.5 μm), a complex mixture, consists mainly of black carbon (BC), soil particles (SOIL), organic matter (OM), sulfate (SO_4_^2−^), ammonium (NH_4_^+^), and nitrate (NO_3_^−^) [6,7]. Previous studies on PM_2.5_ and its constituents have shown increasing evidence of adverse health consequences [8,9,10,11,12,13]. However, the extent of the associations between PM_2.5_ exposure and its constituents and CVD risks varied considerably among different studies. Previous studies have found that inorganic ions such as NH_4_^+^ and SO_4_^2−^ pose the highest risk of CVDs [8,9]. The Chinese Family Panel Study reported that SO_4_^2−^ is the component of PM_2.5_ with the highest risk of CVDs [9]. Another study conducted in China found that NH_4_^+^ presented the highest risk of hospital admissions of CVDs with an excess risk of 2.30% for per interquartile range (IQR) increase [8]. Several studies also indicated that the primary carbon-based fraction was associated with the maximum hazard of CVD compared to other constituents [10,11,12,13,14]. However, another population-based study drawing from 19 cohorts in Europe did not find a significant association of any PM_2.5_ constituents with the mortality of CVDs [15]. These magnitude heterogeneity associations may be explained by variations in the chemical constituents of PM_2.5_. In addition, it is noteworthy that the majority of this research was carried out in economically developed urban areas. Evidence on the associations of PM_2.5_ and its chemical constituents with the risk of ASCVD and the constituents most responsible for these associations in rural areas is lacking.

To date, most existing epidemiological research commonly uses linear or logistic regression models for cross-sectional studies and the Cox regression model for cohort studies to investigate the associations of PM_2.5_ constituent exposure with individuals’ health [16,17,18]. However, these traditional methods failed to assess the effect of redistribution between PM_2.5_ constituents. The constituents available for substitution are heterogeneous and can produce different health effects. Therefore, it is of enormous significance for public health to identify the effect of concentration assigned to different constituents to develop more specific criteria. The isochronous substitution model (ISM), a method that has been universally used to analyze the health effect of redistribution of time of a day [19,20], could provide a more accurate estimate of the potential effects of reallocation between different PM_2.5_ constituents.

In this study, we employed the China-PAR project prediction equation to obtain individuals’ 10-year ASCVD risk [21], which is a prominent early indicator for the onset of ASCVD and has been widely used to explore the association of various factors with the risk of ASCVD [22,23]. Thus, through a cross-sectional design, this study hypothesized that PM_2.5_ and its six constituents are associated with an increased risk of ASCVD and aimed to assess (1) the associations of PM_2.5_ and its six constituents with 10-year ASCVD risk, (2) which constituents are more toxic to 10-year ASCVD, and (3) the impact of reallocation between PM_2.5_ constituents.

## 2. Materials and Methods

### 2.1. Study Population

In this study, participants were enrolled from the baseline survey of the Henan Rural Cohort, and detailed information has been described previously [24]. From 2015 to 2017, 39,259 permanent residents from five regions of Henan province were derived for the baseline survey. Participants were excluded according to the following criteria: (1) missing information on 10-year ASCVD risk predictors (including systolic blood pressure [SBP], waist circumference, total cholesterol [TC], diabetes, high-density lipoprotein cholesterol [HDL-C], and family history of ASCVD) (n = 476); (2) aged <35 years or ≥75 years (n = 3537); and (3) a history of ASCVD (n = 4084). Ultimately, 31,162 participants were finally included. All participants offered written informed consent prior to the survey.

### 2.2. Data Collection and Assessment of 10-Year ASCVD Risk

Baseline characteristics for each participant were acquired using face-to-face interviews, including age, gender (male or female), marital status (married/cohabiting or single/ widowed/divorced/separated), average monthly income (<RMB 500, RMB 500–999, or ≥RMB 1000), educational level (elementary school or below, junior high school or senior high school or above), physical activity (low, moderate or high), smoking and drinking status (never, former or current), high-fat diet (yes or no), and high vegetable and fruit intake (yes or no). Total fat intake and total fruit and vegetable intake were obtained by multiplying the quantity consumed each time by their frequency of consumption. In accordance with the Chinese Residents’ Dietary Guidelines, a high-fat diet and high vegetable and fruit intake are defined as ≥75 g/day and ≥500 g/day [25]. Physical activity levels are evaluated following the International Physical Activity Questionnaire [26].

According to the China ASCVD risk prediction equation, the 10-year ASCVD risk was calculated by the following factors including age, smoking status, waist circumference, treated or untreated systolic blood pressure, family history of ASCVD, TC, HDL-C, diabetes, and region. Waist circumference was assessed twice at 1.0 cm over the umbilicus while wearing light clothing, and the mean of the two assessments was computed for analysis. Blood pressure was monitored three times using an electrophysiological blood pressure monitor (Omron HEM-7071A, Dalian, Liaoning, China), and the mean value was calculated for further analysis. Family history of ASCVD was the occurrence of myocardial infarction, coronary heart disease, or stroke in at least one parent or sibling. Blood samples were taken from participants after a fast of at least 8 h, and blood biochemicals (including TC, fasting glucose, and HDL-C) were measured using an auto-biochemistry analysis system (Cobas c501, Roche, Basel, Switzerland). Diabetes was considered fasting blood glucose ≥7.0 mmol/L, currently receiving anti-diabetic medication, and has self-reported a clinician’s diagnosis of diabetes. In accordance with the China-PAR project, 10-year ASCVD risk values were derived and further grouped into low (<10%) and high (≥10%) [21].

### 2.3. Estimation of PM_2.5_ and Its Constituents

The predicted concentration of six PM_2.5_ constituents (BC, SOIL, OM, SO_4_^2−^, NH_4_^+^, NO_3_^−^) with a resolution of 1 km was calculated by using the GEOS-Chem chemical transport model (CTM) (version 11–01; http://www.geos-chem.org, accessed on 20 March 2022), which used assimilated meteorological and emission inventories as the primary input to simulate the distribution of various constituents [27,28]. Detailed information was described in our previous study [7]. The concentration of PM_2.5_ was conducted by using a hybrid machine learning method, including satellite observations, GEOS-Chem chemical CTM, and a ground-based monitoring method [29]. The 3-year average concentrations of PM_2.5_ and its chemical constituents were evaluated by using the monthly average concentrations of PM_2.5_ and its constituents according to geocoded residential addresses during a 3-year time span prior to the baseline investigation.

### 2.4. Statistical Analysis

Continuous variables were displayed as mean ± standard deviation (SD), and categorical variables were represented as numbers (percentage). Differences in the distribution of PM_2.5_ and its constituents were tested using *t*-tests.

Pearson correlation analysis was used to assess the correlations among PM_2.5_ and its six constituents. To assess the associations of PM_2.5_ and its six chemical constituents with 10-year ASCVD risk and identify more toxic constituents, three methods (constituent concentration, proportion, and residual analyses) were used by means of logistics regressions. Constituent concentration analysis takes the mass and constituent concentrations of PM_2.5_ as independent variables to analyze the relationship between PM_2.5_ and its constituent and ASCVD. Constituent proportion analysis treats constituent proportions as independent variables and adjusts for covariates and PM_2.5_ mass concentrations. Constituent proportion analysis used PM_2.5_ adjusted component concentrations as independent variables and adjusted for covariates. Detailed information is shown in the supplementary material. Two models were constructed below: Model 1 was unadjusted, and Model 2 was adjusted for gender, marital status, educational level, average monthly income, physical activity, high-fat diet, drinking status, and high fruit and vegetable intake. Furthermore, restricted cubic splines were employed to assess the exposure–response relationships. To investigate the susceptible population, stratified analysis was carried out by gender, educational level, average monthly income, physical activity, and high-fat diet. The differences between sub-groups were tested by the significance of the interactions. ISM was utilized to investigate the impact of substitution between PM_2.5_ constituents on 10-year ASCVD risk. In the ISM, one of the constituents was removed, and total PM_2.5_ concentration and other constituents were included to determine the effect of replacing 1 µg/m^3^ of one constituent with 1 µg/m^3^ of another constituent. Detailed information on ISM is presented in the Supplementary Materials. To check the robustness of our results, sensitivity analyses were employed by assessing the relationship between average pollutant exposure concentrations on 5-, 8-, and 10-year time scales and high 10-year ASCVD risk.

Analyses were performed with SPSS version 21.0 (IBM-SPSS Inc., Armonk, NY, USA) and R software version 4.0.3. Two-sided *p*-values less than 0.05 were regarded as statistically significant.

## 3. Results

### 3.1. Characteristics of the Participants

Table 1 summarizes the baseline characteristics. In total, 31,162 participants aged 55.898 ± 9.782 years were included, including 12,163 (39.032%) males and 18,999 (60.968%) females. A total of 8770 of them were determined as having high 10-year ASCVD risk with a prevalence rate of 28.143%. In addition, 4231 (13.577%) participants had a high educational level, 9690 (31.096%) participants had a high average monthly income, 9915 (31.818%) participants had high physical activity, and 6063 (19.456%) participants had a high-fat diet. Supplementary Table S1 displays the summary distribution of PM_2.5_ and its six constituents. The mean (SD) and median (IQR) of the 3-year average PM_2.5_ concentrations were 75.24 (9.600) and 80.160 (16.850) μg/m^3^. Regarding the six PM_2.5_ constituents, the mean (SD) and median (IQR) values of exposure ranged from 5.190 (0.947) and 5.650 (1.920) μg/m^3^ for BC to 18.016 (2.619) and 19.110 (4.120) μg/m^3^ for NO_3_^−^. Correlations between PM_2.5_ and its six constituents are depicted in Supplementary Figure S1. Pearson correlation coefficients ranged from 0.51 to 0.99, which shows high and positive associations with each other. As exhibited in Table 2, mean (SD) of PM_2.5_ (76.813 (9.214) vs. 74.621 (9.681) μg/m^3^), BC (5.368 (0.912) vs. 5.120 (0.951) μg/m^3^), NH_4_^+^ (10.883 (1.368) vs. 10.620 (1.448) μg/m^3^), NIT (18.376 (2.513) vs. 17.875 (2.646) μg/m^3^), OM (15.961 (1.768) vs. 15.540 (1.723) μg/m^3^), SO_4_^2−^(14.851 (1.752) vs. 14.534 (1.848) μg/m^3^), and SOIL (9.984 (1.607) vs. 9.526 (1.550) μg/m^3^) were all higher in high 10-year ASCVD group than in low 10-year ASCVD group (all *p* <0.001).

### 3.2. Associations of PM_2.5_ and Its Six Constituents with 10-Year ASCVD Risk

**Figure 1:**
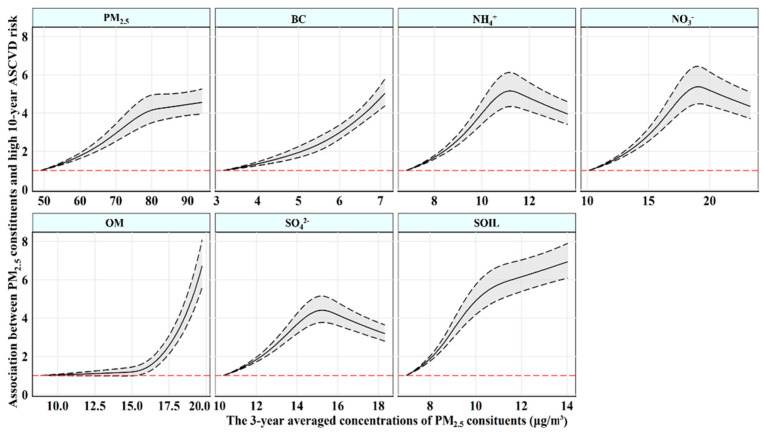
The exposure–response associations of 3-year averaged concentrations of PM_2.5_ constituents with high 10-year ASCVD risk. Abbreviations: PM_2.5_—fine particulate matter; BC—black carbon; NH_4_^+^—ammonium; NO_3_^−^—nitrate; OM—organic matter; SO_4_^2−^—sulfate; SOIL—soil particles; ASCVD—atherosclerotic cardiovascular disease. Models were adjusted for gender, marital status, average monthly income, educational level, physical activity, drinking status, high−fat diet, and fruit and vegetable intake.

Table 3 lists the estimated risk for high 10-year ASCVD risk related to PM_2.5_ and its constituents. After adjusting confounding factors in Model 2, the estimated odds ratio (OR) and 95% confidence interval (95% CI) for 10-year high risk of ASCVD were 1.035 (1.031, 1.038) every 1 µg/m^3^ increase in PM_2.5_. Among the six PM_2.5_ constituents, BC had the strongest association with high 10-year ASCVD risk, with an OR of 1.493 (95% CI: 1.446, 1.542) per 1 µg/m^3^ increase, followed by SOIL (1.285 [95% CI: 1.262, 1.309]), OM (1.214 [95% CI: 1.193, 1.236]), NH_4_^+^ (1.194 [95% CI: 1.170, 1.219]), SO_4_^2−^ (1.142 [95% CI: 1.124, 1.160]), and NO_3_^−^(1.105 [95% CI: 1.092, 1.118]). Comparable results were found in constituent proportion and residual analyses, in which BC was also observed most strongly associated with high 10-year ASCVD risk. In the constituent proportion analyses, the OR and 95% CI for high 10-year ASCVD risk were 3.471 (3.062, 3.935) for each percentile increase in the proportion of BC in PM_2.5_ and 4.554 (3.889, 5.333) per 1 µg/m^3^ increase in the PM_2.5_-adjusted BC concentration in the component residual analysis. Figure 1 depicts the exposure–response associations of the 3-year average concentration of PM_2.5_ and its six constituents with 10-year ASCVD risk, which indicated a linear association between BC and high 10-year ASCVD risk and curvilinear associations of PM_2.5_ and other constituents with high 10-year ASCVD risk. The sensitive analysis for different exposure timescales did not change substantially (Supplementary Figure S2).

### 3.3. Stratified Analyses

**Figure 2:**
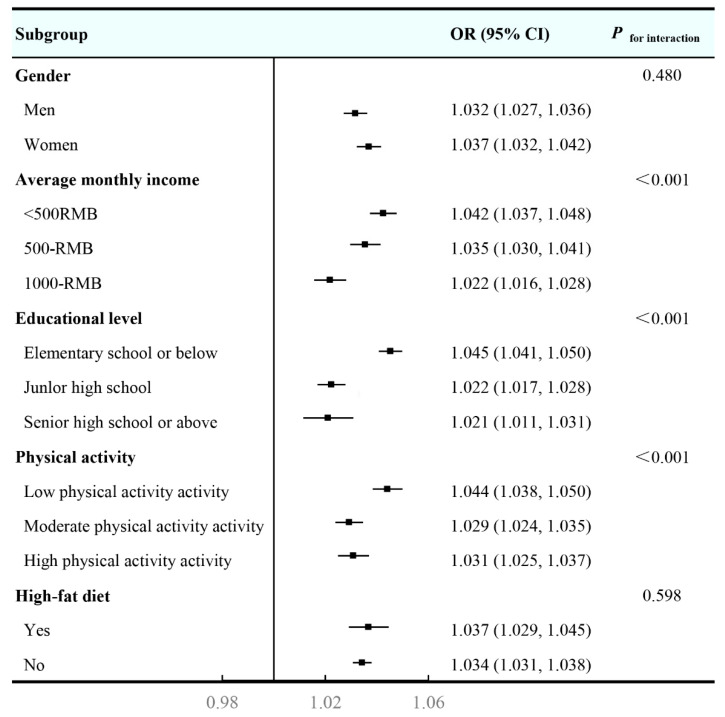
Stratified analyses of the associations between 3-year averaged concentrations of PM_2.5_ with high 10-year ASCVD risk. Abbreviations: PM_2.5_—fine particulate matter; ASCVD—atherosclerotic cardiovascular disease. Models were adjusted for gender, marital status, average monthly income, educational level, physical activity, drinking status, high−fat diet, and fruit and vegetable intake.

**Figure 3:**
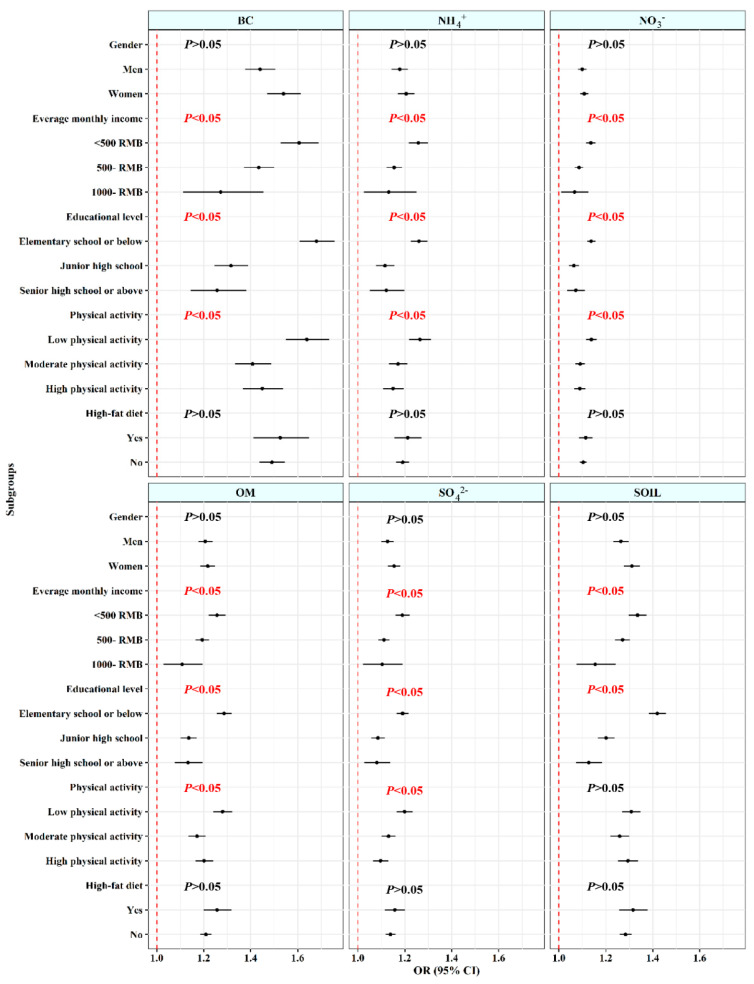
Stratified analyses of the associations between 3-year averaged concentrations of PM_2.5_ constituents with high 10-year ASCVD risk. Abbreviations: PM_2.5_—fine particulate matter; BC—black carbon; NH_4_^+^—ammonium; NO_3_^−^—nitrate; OM—organic matter; SO_4_^2−^—sulfate; SOIL—soil particles; ASCVD—atherosclerotic cardiovascular disease. Models were adjusted for gender, marital status, average monthly income, educational level, physical activity, drinking status, high−fat diet, and fruit and vegetable intake.

Figure 2 illustrates the results of subgroup analyses in multivariable-adjusted constituent concentration models. The results indicated larger estimated effects between PM_2.5_ and 10-year ASCVD risk were observed in those have low average monthly income (OR and 95% CI: 1.042 [1.037, 1.048]), low educational level (OR and 95% CI: 1.045 [1.041, 1.050]), and low physical activity (OR and 95% CI: 1.044 [1.038, 1.050]), with significant interactions between PM_2.5_ and these characteristics (all *p* < 0.001). However, no discernible differences were found for other subgroups. In Figure 3, similar results were also observed in associations of high 10-year ASCVD risk with six PM_2.5_ constituents, wherein no discernible difference was found in the association between SOIL and ASCVD at different levels of physical activity.

### 3.4. Effects of Reallocation between PM_2.5_ Constituents

**Figure 4:**
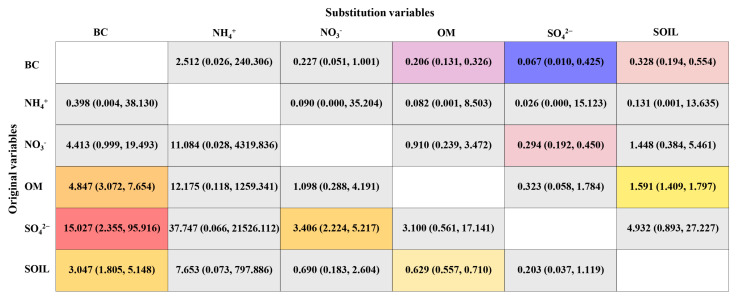
The effects of 1 μg/m^3^ reallocation between BC, NH_4_^+^, NO_3_^−^, OM, SO_4_^2−^, and SOIL on high 10-year ASCVD risk. Abbreviations: OR—odds ratio; 95% CI—95% confidence interval; BC—black carbon; NH_4_^+^—ammonium; NO_3_—nitrate; OM—organic matter; SO_4_^2−^—sulfate; SOIL—soil particles; ASCVD—atherosclerotic cardiovascular disease. Models were adjusted for gender, marital status, average monthly income, educational level, physical activity, drinking status, high−fat diet, and fruit and vegetable intake.

Figure 4 reveals the results of substitution between PM_2.5_ constituents on 10-year ASCVD risk, which indicates that replacing BC with other PM_2.5_ constituents will gain extreme health benefits. The results showed that replacing 1 μg/m^3^ BC with equivalent SO_4_^2−^, SOIL and OM were related to reduced high 10-year ASCVD risk (ORs and 95% CIs: 0.067 [0.010, 0.425] for SO_4_^2−^; 0.328 [0.194, 0.554] for SOIL; and 0.206 [0.131, 0.326] for OM). Whereas replacing 1 μg/m^3^ OM, SO_4_^2−^, and SOIL with equivalent BC were significantly associated with increased high 10-year ASCVD risk (ORs and 95% CIs: 15.027 [2.355, 95.916] for SO_4_^2−^; 4.847 [3.072, 7.654] for OM; and 3.047 [1.805, 5.148] for SOIL). 

## 4. Discussion

Based on a broadscale population health survey, this study first assessed the relationship between PM_2.5_ and six of its constituents with 10-year ASCVD risk by using constituent concentrations, proportion, and residual analysis among rural populations. The results demonstrated that long-term exposure to PM_2.5_ and its constituents are associated with an increased 10-year risk of ASCVD in all three methods. In particular, BC was the most detrimental constituent, and ISM showed that replacing BC with other constituents would yield maximum health benefits. Subgroup analyses indicated that the associations were more pronounced in those with low average monthly income, low educational levels, and low physical activity. These results have critical implications for the establishment of interventions to reduce the risk of ASCVD by reducing PM_2.5_ and especially BC concentrations.

In line with recent studies, we found a significant positive relationship between PM_2.5_ and 10-year ASCVD risk [29,30,31,32,33]. For instance, the NIH-AARP cohort reported that each 10 μg/m^3^ increase in PM_2.5_ corresponded with 16% and 14% higher risks of death from ischemic heart disease and stroke, respectively [34]. Inflammation, oxidative stress, and vascular endothelial dysfunction caused by PM_2.5_ can contribute to the development of atherosclerosis, and previous studies have reported that reducing ambient air pollution concentrations, particularly PM_2.5_, could reduce the ASCVD burden globally [31,35]. Epidemiological evidence on the relationship of PM_2.5_ chemical constituents with 10-year ASCVD risk is limited, whereas researchers have well-reported and demonstrated that long-term exposure to the constituents of PM_2.5_ is positively associated with the risk of CVD, which could support the result of this study indirectly [36,37,38]. A Chinese longitudinal cohort survey reported that per-IQR increases in SO_4_^2−^, NH_4_^+^, NO_3_^−^, and BC were associated with a 72.1%, 53.7%, 31.1%, and 29.4% higher risk of CVD, respectively [9]. In Tibetans of Pakistan, researchers also found significant positive associations between CVD emergency room visits and the concentration of nickel (RR: 1.08, 95% CI: 1.02, 1.15) and NO_3_^−^ (RR: 1.03, 95% CI: 1.00, 1.06). However, no significant association was found between CVD emergency room visits and BC, NH_4_^+^, and NO_3_^−^ [39]. Similar results were also observed in America, which indicated a positive association of PM_2.5_, elemental carbon, and organic carbon with emergency department visits of CVD [40]. However, associations between PM_2.5_ constituents and CVD risks varied across studies, and these discrepancies could be possibly attributed to the composition of the chemical constituents in PM_2.5_ across seasons and regions [41,42].

Our study indicated that the highest risk of ASCVD was associated with the 3-year average concentration of BC exposure, and ISM also revealed that replacing BC with other constituents would reduce the risk of ASCVD. Our results were encoded with previous studies. A previous Chinese cohort study indicated that BC had the strongest association with CVD, ischemic heart disease, and ischemic stroke compared with other constituents [14]. Another cohort study also reported that BC was much more strongly associated with CVD mortality (HR: 1.33 [1.18, 1.50]) than SO_4_^2−^, NO_3_^−^, OC, and NH_4_^+^ (HR: 1.16–1.27) [43]. Furthermore, previous studies also found stronger associations between BC and various health outcomes, including type 2 diabetes mellitus, hypertension in pregnancy, wheezing, and asthma [7,44,45,46]. BC, mainly generated from incomplete combustion of fossil fuels, has been the most investigated PM constituent in previous studies [47,48]. Toxicological researchers have revealed that PM from incomplete combustion is more toxic than complete combustion [49,50].

In stratified analyses, results indicated average monthly income, educational level, and physical activity level may modify the associations between the risk of ASCVD and the 3-year average concentration of PM_2.5_ and its six constituents. Higher estimated OR for high 10-year ASCVD risk were found among those with low average monthly income and low educational level, which is in accordance with previous epidemiological studies [51,52,53,54]. This was probably due to the following reasons: first, population with low average monthly income and low educational level may have more mental stress, which has been demonstrated to be related to the incidence of ASCVD [55]; second, low average monthly income and low educational level group were likely to have fewer medical resources and poorer physical functioning, and were more susceptible to ASCVD when exposed to air pollution [56]; and third, individuals with low average monthly income and low educational level may consume fewer antioxidants (such as vitamins) which can reduce oxidative stress and inflammation caused by PM_2.5_ [57]. In terms of physical activity, it is worth noting that an elevated level of physical activity could significantly minimize the harmful effects of PM_2.5_ and its constituents. In both Korea and China, researchers found consistent results, reporting that a reduction in the level of physical activity could potentially increase PM_2.5_-attributed CVD risks [30,54]. Previous studies have found physical activity could alleviate oxidative stress and systemic inflammation, decelerate atherosclerosis, repair cardiac autonomic nerve dysfunction, and further reduce PM_2.5_-related ASCVD risk [58,59,60].

A handful of potential bio-mechanisms have been raised to clarify the underlying health effects of PM_2.5_, including direct effects, oxidative stress and systemic inflammation, DNA methylation, and disruption of the autonomic nervous system. First, PM_2.5_ and its chemical constituents could cross the pulmonary epithelium and get into the circulation system directly or interact with pulmonary receptors, leading to the production of reactive oxygen species (ROS) and interfering with the modulation of calcium levels, which in turn results in acute cardiovascular injury [61]. Second, inhaled PM_2.5_ constituents, including BC and SO_4_^2−^ can induce oxidative stress and inflammation in the lungs, leading to systemic inflammation, triggering hemostatic circuitry, damaging the functionalities of vessels, and accelerating atherosclerosis [62]. Third, exposure to NH_4_^+^, SO_4_^2−^ and NO_3_^−^ could increase blood pressure by activating the sympathetic–adrenal–medullary and hypothalamic–pituitary–adrenal axes, which may mediate the impact of PM_2.5_ on ASCVD risk [63]. Finally, BC may trigger systemic inflammation through the downregulation of DNA methylation and contribute to the development of cardiovascular disease [64].

This study presents several remarkable strengths. First of all, the data analysis process for this study was based on a large population sample, which was effective in providing comprehensive data and reducing bias. In addition, since the concentration of PM_2.5_ in China is higher than WHO standards and people in rural areas lack social support and medical resources, the Chinese rural population is more vulnerable to PM_2.5_. This study could hint that reducing the concentration of BC may be the key measurement in reducing PM_2.5_-associated ASCVD risks. However, several limitations in this study should be noted. First, individual exposure to air pollution concentrations was estimated based on geocoded residential addresses, which may give rise to spatial misclassification. However, there is evidence that this misclassification effect tends toward zero [65]. Second, the cross-sectional study is limited in explaining causal relationships. However, our study could hint at associations of PM_2.5_ and its constituents with the risk of ASCVD to some degree, and further cohort study is ongoing. Third, exposure concentrations of PM_2.5_ and its constituents were simulated by the Geos-Chem CTM rather than actually measured by ground-based monitors. However, a high degree of cross-validation agreement between the two methods has been reported in a previous study [29].

## 5. Conclusions

In summary, our study provides brand-new evidence on the risk of ASCVD associated with long-term exposure to PM_2.5_ and its six constituents (BC, NO_3_^−^, NH_4_^+^, OM, SO_4_^2−^, and SOIL), in particular BC, functioning as a key element. Stronger PM–ASCVD associations were found in populations with low average monthly income, low educational level, and low physical activity, implying the significance of controlling PM_2.5_ pollution in these susceptible populations.

## Figures and Tables

**Table 1:** Characteristics of the study population.

Variables	All (n = 31,162)
Age (year, mean ± SD)	55.898 ± 9.782
Females (n, %)	18,999 (60.968)
Marital status (n, %)	
Married/cohabitation	28,558 (91.644)
Unmarried/divorced/widowed	2604 (8.356)
Education level (n, %)	
Elementary school or below	13,920 (44.670)
Junior high school	13,011 (41.753)
High school or above	4231 (13.577)
Average monthly income (n, %)	
<RMB 500	10,993 (35.277)
RMB 500–999	10,479 (33.627)
≥RMB 1000	9690 (31.096)
Smoking status (n, %)	
Never	22,727 (72.932)
Ever	2323 (7.455)
Current	6112 (19.614)
Drinking status (n, %)	
Never	24,063 (77.219)
Ever	1259 (4.040)
Current	5840 (18.741)
High-fat diet (yes, n, %)	6063 (19.456)
Adequate vegetable and fruit intake (yes, n, %)	13,221 (42.429)
Physical activity (n, %)	
Low	9360 (30.037)
Moderate	11,887 (38.146)
High	9915 (31.818)
WC (cm, mean ± SD)	84.232 ± 10.209
SBP (mmHg, mean ± SD)	125.566 ± 19.478
DBP (mmHg, mean ± SD)	77.862 ± 11.608
TC (mg/dL, mean ± SD)	184.676 ± 37.361
HDL-C (mg/dL, mean ± SD)	51.287 ± 12.891
Family history of ASCVD (n, %)	4777 (15.330)
Antihypertensive treatment with 2 weeks (n, %)	4365 (76.498)
Diabetes mellitus (n, %)	2824 (9.062)
High 10-year ASCVD risk (n, %)	8770 (28.143)

Abbreviations: ASCVD—atherosclerotic cardiovascular disease; SD—standard deviation; WC—waist circumference; SBP—systolic blood pressure; DBP—diastolic blood pressure; TC—total cholesterol; HDL-C—high-density lipoprotein cholesterol.

**Table 2:** Three-year average concentrations of PM_2.5_ and its constituents according to 10-year ASCVD risk.

Variables	Overall	10-Year ASCVD Risk		*p*-Values *
		Low	High	
PM_2.5_ (μg/m^3^, mean ± SD)	75.238 ± 9.602	74.621 ± 9.681	76.813 ± 9.214	<0.001
PM_2.5_ constituents				
BC (μg/m^3^, mean ± SD)	5.190 ± 0.947	5.120 ± 0.951	5.368 ± 0.912	<0.001
NH_4_^+^ (μg/m^3^, mean ± SD)	10.694 ± 1.431	10.620 ± 1.448	10.883 ± 1.368	<0.001
NIT (μg/m^3^, mean ± SD)	18.016 ± 2.619	17.875 ± 2.646	18.376 ± 2.513	<0.001
OM (μg/m^3^, mean ± SD)	15.659 ± 1.746	15.540 ± 1.723	15.961 ± 1.768	<0.001
SO_4_^2−^ (μg/m^3^, mean ± SD)	14.624 ± 1.827	14.534 ± 1.848	14.851 ± 1.752	<0.001
SOIL (μg/m^3^, mean ± SD)	9.655 ± 1.579	9.526 ± 1.550	9.984 ± 1.607	<0.001

Abbreviations: SD—standard deviation; PM_2.5_—fine particulate matter; BC—black carbon; NH_4_^+^—ammonium; NO_3_^−^—nitrate; OM—organic matter; SO_4_^2−^—sulfate; SOIL—soil particles; ASCVD—atherosclerotic cardiovascular disease. * Student’s *t*-test was used to compare the mean difference in continuous variables between low and high 10-year ASCVD risk groups.

**Table 3:** Associations of PM_2.5_ and its constituents with high 10-year ASCVD risk.

Variables	Logistic Regression ORs (95% CI)	
	Model 1	Model 2
Constituent concentration analyses		
PM_2.5_	1.025 (1.022, 1.028)	1.035 (1.031, 1.038)
PM_2.5_ constituents		
BC	1.328 (1.293, 1.365)	1.493 (1.446, 1.542)
NH_4_^+^	1.139 (1.120, 1.160)	1.194 (1.170, 1.219)
NO_3_^−^	1.078 (1.067, 1.088)	1.105 (1.092, 1.118)
OM	1.160 (1.142, 1.178)	1.214 (1.193, 1.236)
SO_4_^2−^	1.101 (1.086, 1.116)	1.142 (1.124, 1.160)
SOIL	1.196 (1.178, 1.214)	1.285 (1.262, 1.309)
Constituent proportion analyses		
PM_2.5_ constituents		
BC	2.434 (2.180, 2.716)	3.471 (3.062, 3.935)
NH_4_^+^	0.607 (0.568, 0.648)	0.523 (0.486, 0.564)
NO_3_^−^	0.814 (0.788, 0.841)	0.733 (0.706, 0.761)
OM	1.062 (1.034, 1.089)	1.046 (1.016, 1.076)
SO_4_^2−^	0.774 (0.744, 0.805)	0.749 (0.717, 0.783)
SOIL	1.129 (1.111, 1.147)	1.190 (1.169, 1.212)
Constituent residual analyses		
PM_2.5_ constituents		
BC	3.071 (2.672, 3.530)	4.554 (3.889, 5.333)
NH_4_^+^	0.535 (0.493, 0.581)	0.458 (0.417, 0.502)
NO_3_^−^	0.770 (0.737, 0.804)	0.689 (0.656, 0.724)
OM	1.117 (1.080, 1.155)	1.113 (1.073, 1.156)
SO_4_^2−^	0.694 (0.660, 0.730)	0.666 (0.629, 0.704)
SOIL	1.170 (1.145, 1.195)	1.235 (1.206, 1.265)

Abbreviations: OR—odds ratio; 95% CI—95% confidence interval; BC—black carbon; NH_4_^+^—ammonium; NO_3_^−^—nitrate; OM—organic matter; SO_4_^2−^—sulfate; SOIL—soil particles; ASCVD—atherosclerotic cardiovascular disease. Model 1 was the crude model; Model 2 was adjusted for gender, marital status, average monthly income, educational level, physical activity, drinking status, high−fat diet, and fruit and vegetable intake. Concentration: constituent concentration analyses treat PM_2.5_ mass and concentration of components as independent variables and adjust for covariates. Proportion: constituent proportion analyses treat component proportions as independent variables and adjust for covariates and PM_2.5_ mass concentrations. Residual: component proportion analysis treats PM_2.5_ adjusted component concentrations as independent variables and adjusts for covariates.

## Data Availability

The data analyzed during this study are available from the corresponding author upon reasonable request.

## Supplementary Materials

The following supporting information can be downloaded at https://www.mdpi.com/article/10.3390/toxics11100812/s1, Figure S1: Correlation between 3-year averaged concentrations of PM_2.5_ and its constituents; Figure S2: Sensitive analyses of the association between exposure to PM_2.5_ and its constituents and ASCVD risk at different time scales; Table S1: Distributions of 3-year averaged concentrations of PM_2.5_ and its constituents.
