# Supplementary material for: Association of Fine Particulate Matter Constituents with the Predicted 10-Year Atherosclerotic Cardiovascular Disease Risk: Evidence from a Large-Scale Cross-Sectional Study
Source: Toxics. 2023 Sep 26;11(10):812. doi: 10.3390/toxics11100812 (PMC10611010; doi:10.3390/toxics11100812)
Supplement: Supplementary file 1 [file toxics-11-00812-s001.zip › toxics-2618016-supplementary.pdf]

Supplementary appendix

This appendix formed part of the original submission and has been peer reviewed. We post it as supplied by the authors.

Supplement to: Association of $PM_{2.5}$ constituent with the predicted 10-year atherosclerotic cardiovascular disease: evidence from a large-scale rural population study

Authors:

Sheng Wang [1 †], Ge Zhao [1 †], Caiyun Zhang [2], Ning Kang [2], Wei Liao [2], Chongjian Wang [2 *], Fuwei Xie [1,*]

Authors affiliations:

1 Key Laboratory of Tobacco Chemistry, Zhengzhou Tobacco Research Institute of CNTC, Zhengzhou, PR China

2 Department of Epidemiology and Biostatistics, College of Public Health, Zhengzhou University, Zhengzhou, Henan, PR China

* Correspondence: Prof. Chongjian Wang: tjwcj2008@zzu.edu.cn, Dr. Fuwei Xie: xiefuwei@sina.com

† These authors contributed equally to this work.

[*] **Correspondence author**

Dr. Fuwei Xie, and Prof. Chongjian Wang

Key Laboratory of Tobacco Chemistry

Zhengzhou Tobacco Research Institute of CNTC

Fengyang Street #2, Zhengzhou, 450001, Henan, PR China

Phone: +86 371 67781452

Fax: +86 371 67781919

E-mail: xiefuwei@sina.com & tjwcj2008@zzu.edu.cn

Expand methods

Detailed information on the constituent concentration, proportion and residual analyses

The three methods could be expressed as follows:

Logit (P) = $\beta_0 + \beta_1$(constituent or $PM_{2.5}$) + [γ'X] (a)

Logit (P) = $\beta_0 + \beta_1$(constituent/$PM_{2.5}$) +β_2($PM_{2.5}$) + [γ'X] (b)

Logit (P) = $\beta_0 + \beta_1$(residual) + [γ'X] (c)

Formula (a) was constituent concentration analyses, formula (b) was constituent proportion analyses, and formula (c) was constituent residual analyses. P represented the probability of a positive result occurring. β_0 was the intercept, β_1 was the coefficient of exposure to $PM_{2.5}$ or its constituent and [γ'X] was the matrix of other covariates, residual reflected the levels of variation in the constituents, which were independent of $PM_{2.5}$ and was calculated as the residuals of linear regression models: consistent = $\beta_0 + \beta_1$ ($PM_{2.5}$).

Detailed information on the isochronous substitution model (ISM)

In the ISM with one of the constituents removed, total $PM_{2.5}$ concentration and other constituents were included in this model to determine the effect of replacing 1 μg/m^3 of one constituent with equivalent another constituent through logistic regression models. For example, when we calculated the effect of replacing 1 μg/m^3 other constituents with BC, the formula was as follows:

logit(P) = $\beta_0 + \beta_1(NH_4^+) + \beta_2(NO_3^-) + \beta_3$(OM) + $\beta_4(SO_4^{2-})$ +β_5(SOIL) + β_6(Other) + β_7($PM_{2.5}$) + [γ'X]

P represented the probability of high 10-year ASCVD risk occurring. β_0 was the intercept. β_1 was the effect of replacing 1 μg/m^3 NH_4^+ with 1 μg/m^3 BC. By parity of reasoning, β_2 represents the effect of replacing 1 μg/m^3 NO_3^- with 1 μg/m^3 BC. The concentration of other constituent was calculated by subtracting the concentration of BC, NH_4^+, NO_3^-, OM, SO_4^{2-} and SOIL from the concentration of $PM_{2.5}$. [γ'X] was matrix of other covariates.

Table Legend

Supplementary table S1. Distributions of 3-year averaged concentrations of $PM_{2.5}$ and its constituents.

Figure Legends

Supplementary Figure S1. Correlation between 3-year averaged concentrations of $PM_{2.5}$ and its constituents.

Supplementary Figure S2. Sensitive analyses of the association between exposure to PM2.5 and its constituents and ASCVD risk at different time scales.

Supplementary table S1. Distributions of 3-year averaged concentrations of $PM_{2.5}$ and its constituents.

Variables	Mean ± SD	Min	Percentiles			Max	IQR
			P_{25}	P_{50}	P_{75}		
$PM_{2.5}$ ($\mu g/m^3$)	75.24 ± 9.600	48.970	65.960	80.160	82.810	94.090	16.850
Constituents							
BC ($\mu g/m^3$)	5.190 ± 0.947	3.160	4.200	5.650	6.120	7.110	1.920
NH_4^+ ($\mu g/m^3$)	10.694 ± 1.431	6.800	9.300	11.170	11.730	13.620	2.430
NO_3^- ($\mu g/m^3$)	18.016 ± 2.619	10.120	15.720	19.110	19.840	23.330	4.120
OM ($\mu g/m^3$)	15.659 ± 1.746	8.840	14.330	15.710	17.210	19.730	2.880
SO_4^{2-} ($\mu g/m^3$)	14.624 ± 1.827	10.370	12.640	15.190	16.080	18.320	3.440
SOIL ($\mu g/m^3$)	9.655 ± 1.579	6.940	8.360	9.130	11.760	14.030	3.400

Abbreviation: $PM_{2.5}$, fine particulate matter; BC, black carbon; NH_4^+, ammonium; NO_3^-, nitrate; OM, organic matter; SO_4^{2-}, sulfate; SOIL, soil particles.

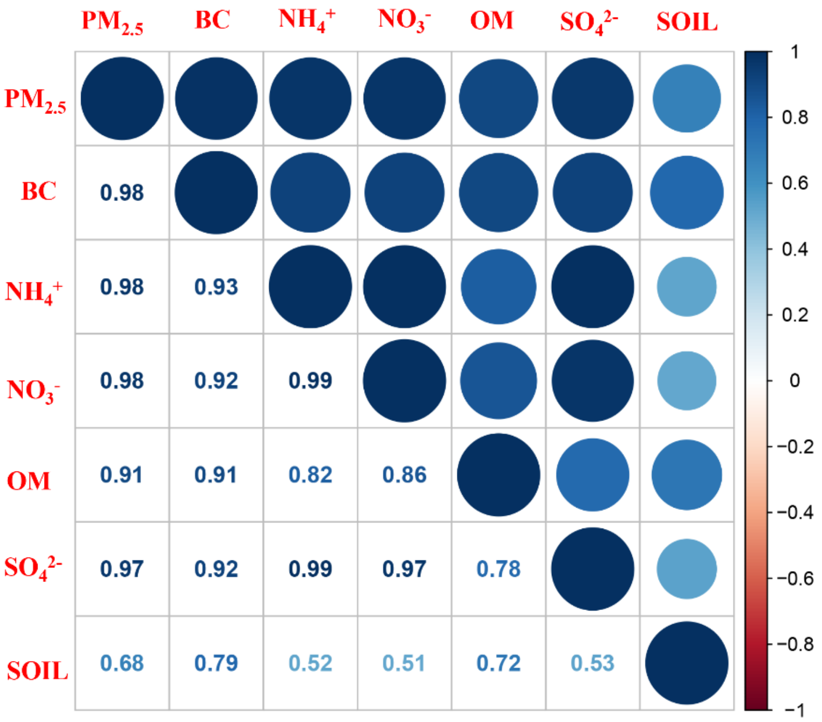

Supplementary Figure S1. Correlation between 3-year averaged concentrations of $PM_{2.5}$ and its constituents.

Abbreviation: $PM_{2.5}$, fine particulate matter; BC, black carbon; NH_4^+, ammonium; NO_3^-, nitrate; OM, organic matter; SO_4^{2-}, sulfate; SOIL, soil particles.

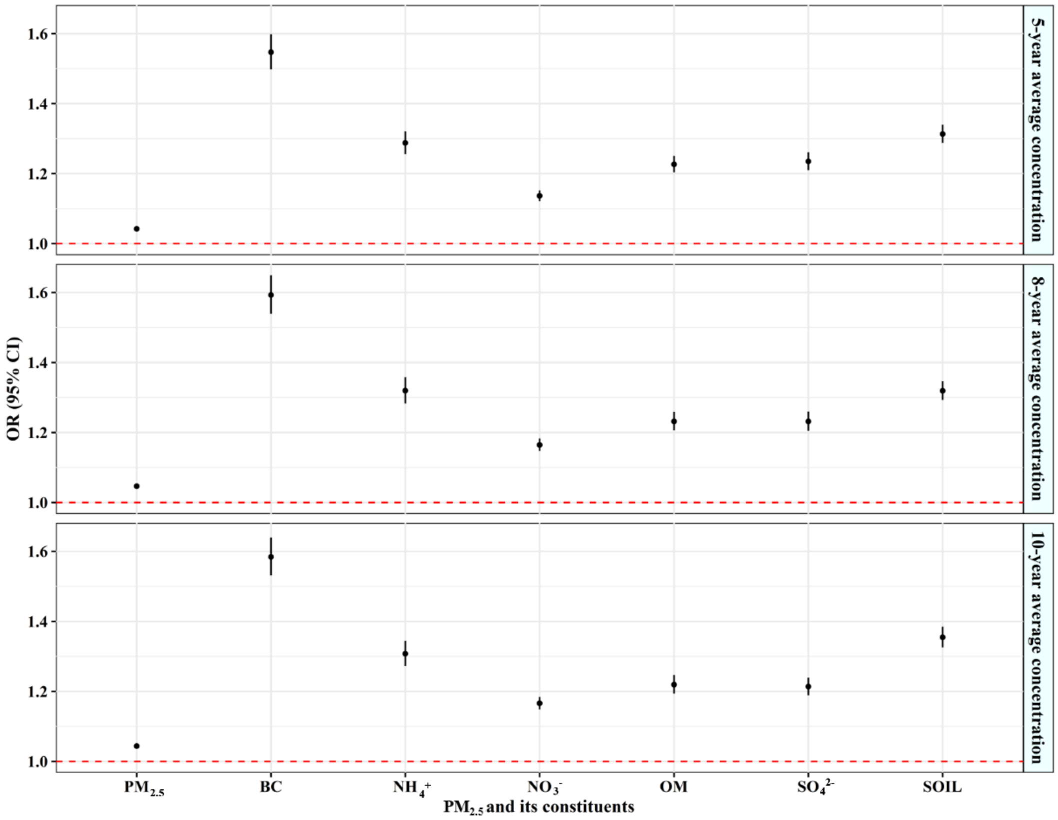

Supplementary Figure S2. Sensitive analyses of the association between exposure to $PM_{2.5}$ and its constituents and ASCVD risk at different time scales.

Abbreviation: $PM_{2.5}$, fine particulate matter; BC, black carbon; NH_4^+, ammonium; NO_3^-, nitrate; OM, organic matter; SO_4^{2-}, sulfate; SOIL, soil particles; ASCVD: atherosclerotic cardiovascular disease.

Models were adjusted for gender, marital status, average monthly income, educational level, physical activity, drinking status, high fat diet, fruit and vegetable intake.
